# Altered mRNA Expression of Interleukin-1 Receptors in Myocardial Tissue of Patients with Left Ventricular Assist Device Support

**DOI:** 10.3390/jcm10214856

**Published:** 2021-10-22

**Authors:** Naima Niazy, Linus Mrozek, Mareike Barth, Moritz Benjamin Immohr, Nikolaos Kalampokas, Diyar Saeed, Hug Aubin, Yukiharu Sugimura, Ralf Westenfeld, Udo Boeken, Artur Lichtenberg, Payam Akhyari

**Affiliations:** 1 Department of Cardiac Surgery, Medical Faculty and University Hospital Düsseldorf, Heinrich-Heine-University Düsseldorf, 40225 Düsseldorf, Germany; Naima.Niazy@med.uni-duesseldorf.de (N.N.); Linus.Mrozek@med.uni-duesseldorf.de (L.M.); Mareike.Barth@med.uni-duesseldorf.de (M.B.); Moritz.Immohr@med.uni-duesseldorf.de (M.B.I.); Nikolaos.Kalampokas@med.uni-duesseldorf.de (N.K.); Diyar.Saeed@med.uni-duesseldorf.de (D.S.); Hug.Aubin@med.uni-duesseldorf.de (H.A.); Yukiharu.Sugimura@med.uni-duesseldorf.de (Y.S.); Udo.Boeken@med.uni-duesseldorf.de (U.B.); Payam.Akhyari@med.uni-duesseldorf.de (P.A.); 2 Department of Cardiac Surgery, Leipzig Heart Center, 04289 Leipzig, Germany; 3 Department of Cardiology, Pneumology and Angiology, Medical Faculty and University Hospital Düsseldorf, Heinrich-Heine-University Düsseldorf, 40225 Düsseldorf, Germany; Ralf.Westenfeld@med.uni-duesseldorf.de

**Keywords:** heart failure, interleukin 1 beta, interleukin 33, mechanical circulatory support

## Abstract

Serum levels of cytokines interleukin 1 beta ( *IL-1β*) and interleukin 33 (IL-33) are highly abnormal in heart failure and remain elevated after mechanical circulatory support (MCS). However, local cytokine signaling induction remains elusive. Left (LV) and right ventricular (RV) myocardial tissue specimens of end-stage heart failure (HF) patients without (*n* = 24) and with MCS (*n* = 39; 594 ± 57 days) were analyzed for cytokine mRNA expression level of *IL-1B*, interleukin 1 receptor 1/2 (*IL-1R1/2*), interleukin 1 receptor-like 1 (*IL-1RL1*), *IL-33* and interleukin-1 receptor accessory protein (*IL-1RaP*). MCS patients showed significantly elevated *IL-1B* expression levels (LV: 2.0 fold, *p* = 0.0058; RV: 3.3 fold, *p* < 0.0001). Moreover, *IL-1R1*, *IL-1RaP* and *IL-33* expression levels strongly correlated with each other. IL-1RL1 and *IL-1R2* expression levels were significantly higher in RV myocardial tissue (RV/LV ratio *IL-1R2* HF: 4.400 ± 1.359; MCS: 4.657 ± 0.655; IL-1RL1 HF: 3.697 ± 0.876; MCS: 4.529 ± 0.5839). In addition, IL1-RaP and *IL-33* RV expression levels were significantly elevated in MCS. Furthermore, *IL-33* expression correlates with C-reactive protein (CRP) plasma levels in HF, but not in MCS patients. Increased expression of *IL-1B* and altered correlation patterns of *IL-1* receptors indicate enhanced *IL-1β* signaling in MCS patients. Correlation of *IL-1* receptor expression with *IL-33* may hint towards a link between both pathways. Moreover, diverging expression in LV and RV suggests specific regulation of local cytokine signaling.

## 1. Introduction

Heart failure (HF) is a major cause of mortality and morbidity in industrialized nations [1]. Due to the ongoing shortage of available donor organs, mechanical circulatory support (MCS) devices have been increasingly utilized for managing HF as a bridge to transplantation or destination therapy [1,2]. The use of left ventricular assist devices (LVADs) leads to decreased mortality and improved quality of life. However, MCS may be associated with device-related complications, such as infection, thromboembolic events and bleeding complications. Further, recent evidence suggests an increase in general systemic inflammation levels in patients with MCS [2,3].

Interleukin 1 beta ( *IL-1β*) is a potent pro-inflammatory mediator that has been correlated to a number of diseases like myocardial infarction, cardiomyopathy and acute myocarditis [4,5,6,7]. *IL-1β* signaling is involved in inflammation and the fibrotic remodeling of heart tissue [8,9,10]. Plasma and myocardial expression levels of *IL-1β* are elevated in end stage HF and remain high after the implantation of MCS devices [11,12]. The constitutively expressed *IL-1* type 1 receptor (*IL-1R1*) and its co-receptor, interleukin-1 receptor accessory protein (*IL-1RaP*), mediate effects of *IL-1β* signaling. The decoy receptor, *IL-1* receptor type 2 (*IL-1R2*), competitively inhibits signaling by binding *IL-1β* without signal transduction [13].

In contrast, *IL-1* family member, interleukin 33 (*IL-33*), likely has a cardio-protective function in the context of HF [14,15], but may also aggravate cardiac inflammation [16]. *IL-33* is rapidly released from cells during necrosis or tissue injury and has been shown to inhibit cardiomyocyte hypertrophy, fibrosis, and apoptosis [14,17]. *IL-33* binds to interleukin 1 receptor-like 1 (*IL-1RL1*), also known as suppression of tumorigenicity-2 (ST2), which exists in two major isoforms: membrane-bound and truncated soluble form (sST2) [18]. Circulating sST2 levels are associated with the risk of cardiovascular death or worsened HF, making sST2 a promising prognostic biomarker for HF [19,20,21]. Although sST2 likely acts as a decoy receptor for *IL-33* [15], the role of *IL-33* signaling in HF has not been fully elucidated. sST2 plasma levels do not correlate with tissue expression of ST2 [22], and previous studies have suggested that the main source for elevated plasma levels of *IL-33* and sST2 are vascular endothelial cells [23,24], indicating that global cytokine levels are regulated, regardless of local signaling within the heart. While serum levels of *IL-1* and *IL-33* signaling molecules have been addressed in several studies [25,26,27], local regulation of receptors within the heart remains elusive. To shed light on local regulation in patients with HF, we analyzed myocardial tissue expression levels of *IL-1* and *IL-33* receptors and co-receptors in this study. Following the hypothesis that LVAD support is associated with alterations of local inflammatory pathways [3], we compared interleukin expression levels in the myocardium of patients with and without MCS. Assuming topographic differences, we examined left (LV) and right ventricular (RV) tissue.

## 2. Materials and Methods

### 2.1. Study Design

This is a retrospective study utilizing myocardial samples obtained between August 2011 and December 2018 undergoing heart transplantation (HTx). LV and RV myocardial tissue samples were collected at the time of HTx, shock frozen and stored in liquid nitrogen. The entire study population included 101 patients. Patients with significant medical history, including cancer or infectious diseases, were excluded, and clinical data from remaining 63 patients were analyzed retrospectively. Exclusion criteria amongst others were short LVAD implantation period, HF causes other than ischemic cardiomyopathy (ICM) or non-ischemic dilated cardiomyopathy (DCM), and previously otherwise analyzed myocardial tissue samples (see also Figure S1). The finally analyzed cohort contained 24 patients with HF who did not require LVAD assistance and 39 patients with MCS. Clinical data, including demographics, medications, comorbidities, and laboratory data, were collected prior to cardiac transplantation and are displayed in Table 1. Twenty-five patients were diagnosed with ICM and 38 patients with DCM. An overview on study selection criteria is given in Figure S1. The study protocol was approved by the ethics committee of the Heinrich-Heine-University (No. 4567) and conforms to the principles outlined in the Declaration of Helsinki. Written informed consent was obtained from all individuals prior to inclusion into the study.

### 2.2. mRNA-Isolation and Real-Time PCR

In the following, gene names are written in italics. In the case of *IL-1β*, *IL-1B* refers to the corresponding gene name. Tissue samples were homogenized and mRNA was isolated using trizol-chloroform extraction followed by mRNA precipitation out of the aqueous phase (TRI Reagent from Sigma-Aldrich, Munich, Germany; protocol according to manufacturer’s instruction). Further purification of mRNA and reverse transcription were performed using Qiagen RNeasy Mini Kit (Qiagen, Hilden, NRW, Germany) and QuantiTectR Reverse Transcription Kit (Qiagen, Hilden, NRW, Germany) according to manufacturer’s specifications. Real-time PCR-Mix contained GoTaqR real-time PCR Master Mix (Promega, Madison, Wisconsin, USA) containing SYBR green as fluorophore and CXR as reference dye, cDNA (10 ng) and exon spanning forward (fw) and reverse (rv) primers (*RPL13A* fw: GTACGCTGTGAAGGCATCAA, rv: GTTGGTGTTCATCCGCTTG, *IL-1B* fw: AGCTGATGGCCCTAAACAGA, rv: GGAGATTCGTAGCTGGATGC; *IL-1R1* fw: TGTGATTGTGAGCCCAGCTA, rv: ACTGACCCATTCCACTTCCA, *IL-1R2* fw: TGAAGGCCAGCAATACAACA, rv: CTTGACCCCAGAGAAGCTGA, *IL-1RaP* fw: TGTCAAACCGACTATCACTTGG, rv: TTGAAATTAAGGCAATGAGGAAA, *IL-33* fw: TGCCAACAACAAGGAACACT, rv: AGGACAAAGAAGGCCTGGTC, *IL-1RL1* fw: TGTGGCAGCTTAATGGAACA, rv: TCTAGACAAGCCAGCCCATT, final concentration 0.15 mM). Real-time quantitative amplification of mRNA was performed according to the manufacturer’s instructions in a StepOnePlus Real Time PCR System (Applied Biosystems Inc, Waltham, MA, USA) in duplicates. Specificity of obtained real-time quantitative PCR products was checked via melting curve analysis (Figure S2). Fold change of gene expression levels was calculated using comparative ∆∆CT method with *RPL13A* as reference gene.

### 2.3. Statistical Analysis

Significance of differences within the study population was tested using Mann-Whitney U test or (for dichotome values) two-sided Fisher’s exact test. Relative mRNA expression levels were presented as boxplots and whisker plots, and significant differences between groups were determined with nonparametric testing (Kruskal-Wallis Test and Dunn’s post-hoc test). Differences in gene expression of RV and LV myocardia within the same patient was analyzed with Wilcoxon signed-rank test. Data are reported as mean ± standard error mean (SEM). *p*-values ≤ 0.05 were considered statistically significant. All datasets were analyzed using GraphPad Prism version 5.01 for Windows (GraphPad Software, La Jolla, California, USA) and IBM SPSS Statistics Version 25.0.0.2 for Windows (IBM Corp. Armonk, NY, USA).

## 3. Results

### 3.1. Patient Demographics

Patients’ ages at transplantation ranged from 33 to 70 years (mean 54 years), and 50 out of 63 patients (79%) were male. 24 patients had HF, and 39 patients received MCS. Patients with MCS prior to HTx were supported for at least 90 days, with a mean duration of 594 days ± 57 days. The majority (27 patients, 69%) were supported with a HeartWare, 8 patients (21%) with a HeartMate II and 4 patients (10%) with a HeartMate III. Nine patients (23%) received an additional right ventricular assist device (RVAD) or an extracorporeal life support system (ECLS) within the period of LVAD support. Demographics are summarized in Table 2. Patient groups were comparable regarding gender, age, body mass index (BMI), ejection fraction, comorbidities and most laboratory values. N-terminal prohormone of brain natriuretic peptide (NTproBNP) levels were elevated in both patient groups, but were significantly higher in HF patients (*p* = 0.002). In addition, the New York Heart Association classification (NYHA) was significantly altered between groups (*p* = 0.0001). The majority of MCS patients was treated with antiplatelet agents (MCS: 32 (82%), HF: 9 (38%), *p* = 0.001) and phosphodiesterase-5 inhibitors (MCS: 22 (56%), HF: 4 (10%), *p* = 0.003). In contrast, HF patients were more frequently treated with antiarrhythmic therapy (HF: 12 (50%), MCS: 8 (21%), *p* = 0.001), while patients with MCS weretreated with calcium antagonists (MCS: 10 (26%), HF: 1 (4%), *p* = 0.04).

### 3.2. Increased Gene Expression of *IL-1B* in Patients with MCS

**Figure 1:**
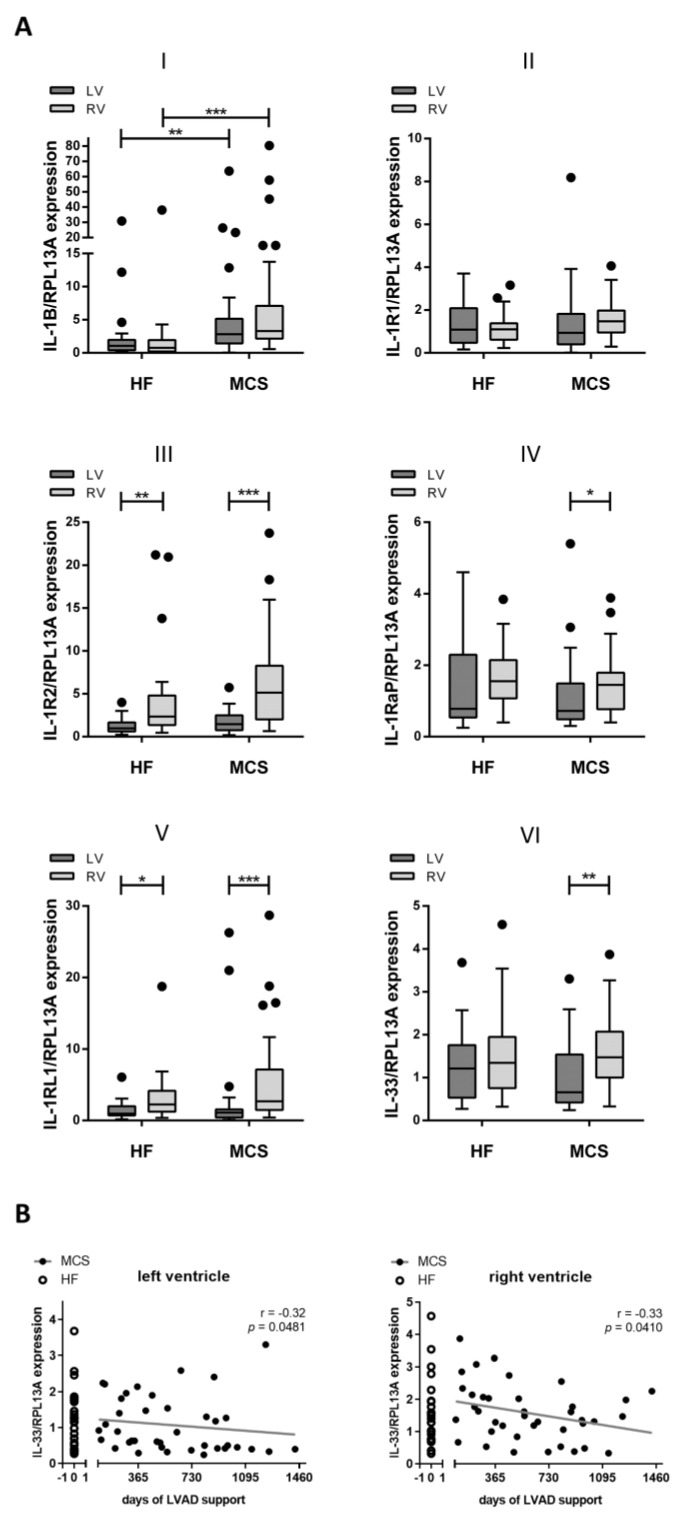
Significantly elevated *IL-1B* expression due to MCS (mechanical circulatory support). (**A**) Fold change of (I) *IL-1B*, (II) *IL-1R1*, (III) *IL-1R2*, (IV) *IL-1RaP*, (V) *IL-1RL1* and (VI) *IL-33* mRNA expression levels are displayed for patients with heart failure (HF) or MCS in left (LV) and right ventricular (RV) myocardial tissue. Data are presented as boxplots with whiskers (Tukey method). Significance of differences is calculated with Kruskal-Wallis-Test and Dunn’s post-hoc test and indicated with asterisks (* *p* < 0.05, ** *p* < 0.01, *** *p* < 0.001). (**B**) *IL-33* expression levels are plotted against MCS duration and analyzed with Spearman correlation. Regression line is indicated in grey. VAD, ventricular assist device.

To shed light on the role of *IL-1β* and *IL-33* signaling within the myocardium of heart failure patients, the expression levels of *IL-1B*, *IL-1* receptors *IL-1R1*, *IL-1R2* and *IL-1RaP* and of *IL-33* and *IL-1RL1* were determined via real-time quantitative PCR analysis (Figure 1). The LV and RV myocardial tissues of patients with and without MCS were analyzed. Expression levels of the *IL-1* receptor antagonist ranged at the level of the analytical detection limit and were, therefore, not included in further analysis (data not shown). *IL-1B* expression levels were significantly higher in the MCS group compared to the HF group (LV: 2.0 fold, *p* = 0.0058; RV: 3.3 fold, *p* < 0.0001; Figure 1A(I)). This applied to both the LV and RV myocardial tissues. While MCS was not associated with changes in *IL-1R2* and *IL1-RL1* expression, comparing LV and RV specimens resulted in significantly altered gene expression (Figure 1A(III,V)). Moreover, expression of *IL1-RaP* and *IL-33* was increased in right ventricular specimens of MCS patients, compared to left ventricle specimens, but was unaltered between ventricles in HF patients (Figure 1A(IV,VI)). mRNA expression levels of *IL-1R1* (Figure 1A(II)) showed no significant differences in patients due to MCS or between ventricles.

In addition, we calculated the Spearman correlation of MCS duration with gene expression. *IL-33* gene expression showed a moderate negative correlation with the duration of LVAD support in both ventricles (LV: r = −0.32, RV: r = −0.33; Figure 1B). However, the observed effect remained within the scattering of *IL-33* gene expression levels that were observed in the HF group.

### 3.3. Correlation of *IL-1* Receptor and *IL-33* Expression

Next, we analyzed whether a correlation of receptor gene expression within the examined pathways existed. Unsurprisingly, strong and highly significant correlations of *IL-1R1* mRNA expression and its co-receptor, *IL-1RaP*, were detectable in all conditions (r ≥ 0.73, *p* < 0.0001; Table 2). Interestingly, correlation patterns indicated a crosslink between *IL-1* receptors and *IL-33*. Both *IL-1R1* and *IL-1RaP* expression showed a strong, highly significant correlation with *IL-33* expression (r ≥ 0.57, *p* ≤ 0.0001). In addition, expression of *IL-1R1* and *IL-33* receptor *IL-1RL1* significantly correlated in both ventricles and all patient groups (r ≥ 0.36, *p* ≤ 0.0224). Moreover, the correlation of *IL-1RaP* and *IL-1RL1* was significant in HF patients (LV: r = 0.46, *p* = 0.0248, RV: r = 0.68, *p* = 0.0003). In contrast, the correlation of *IL-33* and its receptor, *IL-1RL1*, was only observed in the RV tissue of HF patients (r = 0.58, *p* = 0.0031), but was not significant in the other conditions. Overall results indicate the correlation of *IL-1* receptors to *IL-33* signaling.

### 3.4. Altered Expression and Correlation Patterns in Right versus Left Ventricle

**Figure 2:**
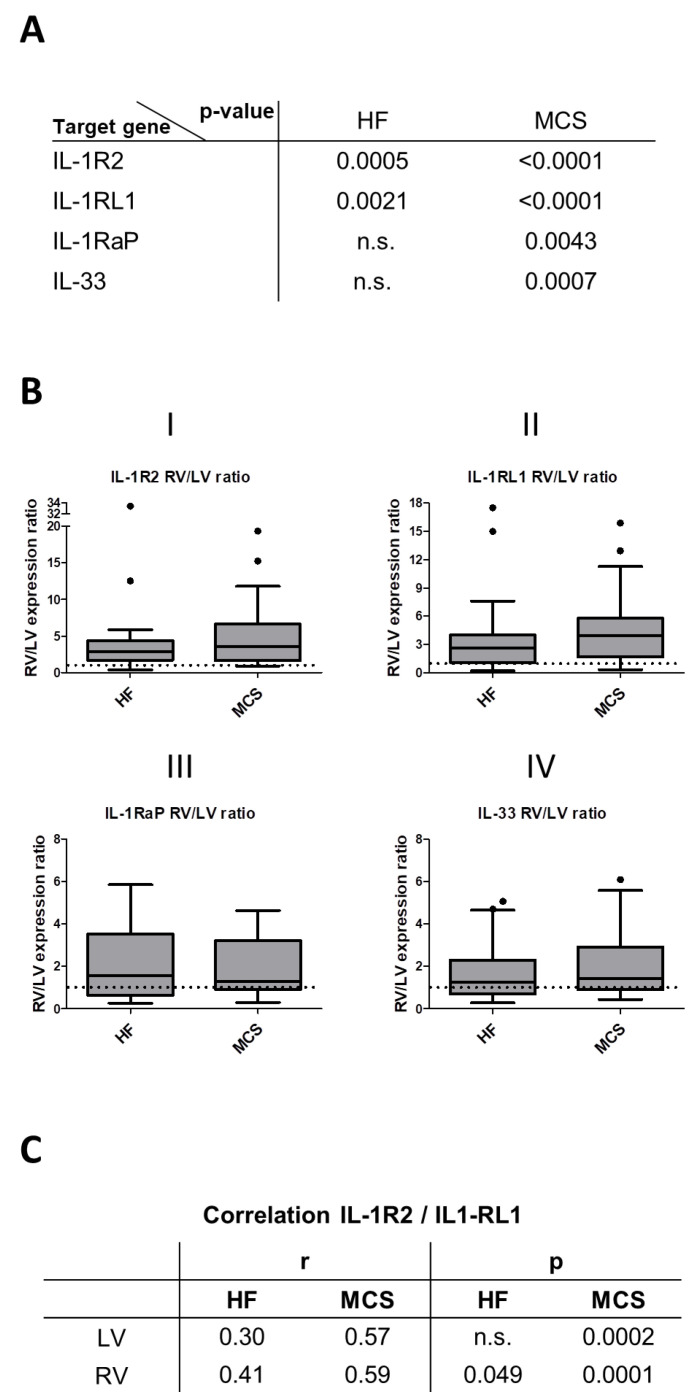
Elevation of gene expression in right ventricular tissue. (**A**) *p*-values of Wilcoxon matched-pairs signed-rank test comparing left versus right ventricular expression within the same patient is displayed. (**B**) Ratio of right ventricular (RV) to left ventricular (LV) mRNA expression levels of (I) *IL-1R2*, (II) *IL-1RL1*, (III) *IL-1RaP* and (IV) *IL-33* are calculated. Values higher than one (dotted line) indicate elevated expression in RV. (**C**) Spearman correlation (r) of *IL-1R2* with *IL-1RL1* is calculated as displayed.

As indicated further above, RV myocardial tissue showed a significantly higher expression of *IL-1R2* and *IL1RL1* (Figure 1A(III,V)). Significant differences within the same patient were confirmed with Wilcoxon matched-pairs signed-rank test in both the HF and the MCS groups (*p*-values displayed in Figure 2A). For further validation, the effect-size RV/LV expression ratio was determined (Figure 2B). The mean RV/LV expression ratio indicated a fourfold higher RV expression of *IL-1R2* (HF: 4.400 ± 1.359; MCS: 4.657 ± 0.655, Figure 2B (I)) and *IL-1RL1* (HF: 3.697 ± 0.876; MCS: 4.529 ± 0.584, Figure 2B(II)). The number of patients with an RV/LV expression ratio lower than one was 17% in HF and 3% in the MCS group for both genes. Moreover, right ventricular *IL-1R2* mRNA levels were elevated in MCS patients by a strong trend (*p* = 0.056), while left ventricular expression remained unaffected (*p* = 0.540). In addition to higher RV mRNA expression levels of *IL-1R2* and *IL-1RL1*, expression of both genes correlated in RV HF and in patients with MCS (*p*-values displayed in Figure 2C).

Furthermore, higher expression of *IL1-RaP* and *IL-33* in RV was confirmed by the following results: Wilcoxon matched-pairs signed-rank test results remained not significant for HF, but highly significant for MCS (Figure 2A). The mean RV/LV expression ratio showed twofold higher RV expression of *IL1-RaP* (HF: 2.113 ± 0.369; MCS: 1.964 ± 0.2156, Figure 2B(III)) and *IL-33* (HF: 1.734 ± 0.2910; MCS: 2.062 ± 0.258, Figure 2B(IV)). Therefore, expression levels were affected specifically by the topographic origin of the analyzed myocardial tissue.

### 3.5. Correlation with CRP Plasma Levels and Leucocyte Count

To validate whether an association of local expression with systemic levels of inflammation exists, gene expression levels were correlated with CRP serum levels and circulating leucocyte counts (Table 3). The correlation of CRP serum levels with gene expression was observed in both ventricles and patient groups, while the correlation with leucocyte counts was only observed in the LV tissue of HF patients. The Spearman correlation of *IL-1R1* with CRP-serum levels was significant in the LV of HF patients (r = 0.56) and with a strong tendency in the LV of MCS patients (r = 0.32, *p* = 0.053). *IL-1R1* also correlated with leucocyte counts in the LV of HF patients (r = 0.52). Further, *IL-33* expression correlated significantly with the CRP levels in both ventricles of HF patients (LV: r = 0.58, RV: r = 0.48) and the LV of HF patients (r = 0.46). Some other receptors investigated here showed weak or no association of their expression with CRP levels or leucocyte counts, indicating a correlation of systemic inflammation markers and local gene expression levels for some, but not all, analyzed markers (see Table 3).

### 3.6. Differences between Patients with ICM and DCM

Since *IL-1β* and *IL-33* signaling impacts on wound repair and fibrosis after myocardial infarction, we divided our patient cohort into patients with ICM and DCM. The group with DCM was younger (DCM: 50 ± 1.8 years, ICM: 60 ± 1.4 years, *p* = 0.02) and had, on average, a lower NYHA class (*p* = 0.014), but did not significantly differ in gender, BMI or LV ejection fraction. Hypertension (DCM: 14 (37%), ICM: 16 (64%), *p* = 0.044), diabetes (DCM: 6 (16%), ICM: 15 (60%), *p* = 0.0001) and treatment with antidiabetic agents (DCM: 2 (5%), ICM: 7 (28%), *p* = 0.023) were less common in the DCM group, as well as treatment with statins (DCM: 13 (34%), ICM: 17 (68%), *p* = 0.011). Other comorbidities, medical treatments and laboratory data did not significantly differ between the ICM and DCM groups (see Table S1).

**Figure 3:**
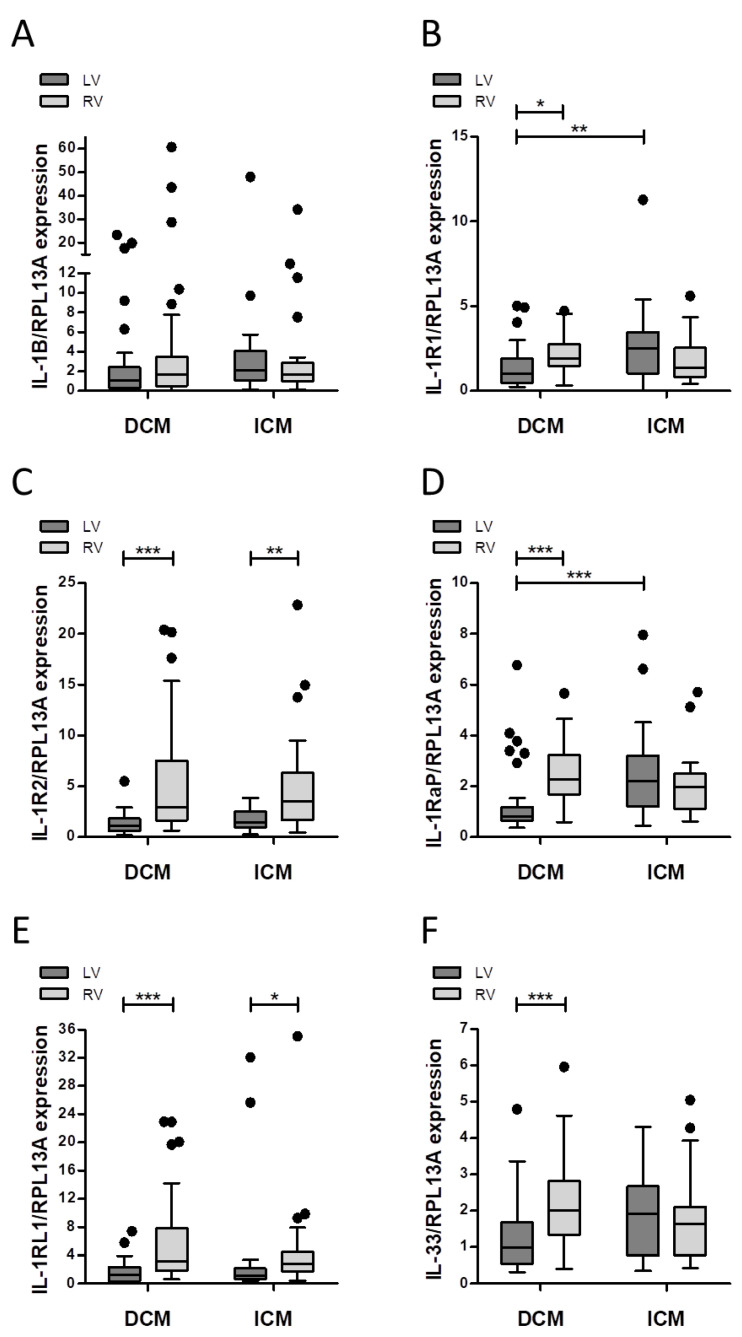
Decreased left ventricular expression of *IL-1R1* and *IL-1RaP* in dilated cardiomyopathy (DCM) versus ischemic cardiomyopathy (ICM). Expression levels of (**A**) *IL-1B*, (**B**) *IL-1R1*, (**C**) *IL-1R2*, (**D**) *IL-1RaP*, (**E**) *IL-1RL1* and (**F**) *IL-33* are compared for left (LV) and right ventricular (RV) myocardium of patients with heart failure (HF) or left ventricular assist devices (LVAD) support. Data are presented as boxplots with whiskers (Tukey method). Data were analyzed with Kruskal-Wallis Test and Dunn’s post-hoc test (* *p* < 0.05, ** *p* < 0.01, *** *p* < 0.001).

Analysis of mRNA expression levels (Figure 3) showed significantly lower levels of *IL-1R1* in the LV myocardial tissue of DCM patients, compared to the RV of both DCM and ICM patients (Figure 3B). Similar effects were observed for *IL-1RaP* expression (Figure 3D). Furthermore, *IL-33* expression was significantly lower in the LV tissue of DCM patients, compared to the RV, but no significant differences between the DCM and ICM groups existed (Figure 3F). In summary, expression patterns were altered depending on the type of cardiomyopathy.

## 4. Discussion

Inflammatory mediators contribute to the development and progression of HF and are associated with the deterioration of cardiac function. Although MCS improves functional capacity and overall survival [28], systemic inflammation levels remain highly elevated [3]. The implications of a persistent inflammatory response remain unclear, highlighting the importance of determining the impact of MCS on pro-inflammatory signaling. The herein presented study investigated the local expression of *IL-1B* and *IL-33* and their respective receptors in HF and MCS patient groups undergoing HTx. Our results demonstrate that *IL-1B* gene expression is significantly higher in patients with MCS than in HF patients. Previous work in this field has demonstrated an upregulation of *IL-1β* in heart failure patients, compared to non-failing controls [12,29], and even higher levels in deteriorating patients requiring LVAD implantation [11,30]. Longitudinal data have shown no significant changes in the first three months (89 ± 66 days) of LVAD support [12]. In contrast, our data, derived from a cohort with a mean LVAD support of 594 ± 57 days, demonstrates a clear elevation of *IL-1B* levels, indicating that *IL-1B* upregulation may occur later. Moreover, in MCS, *IL-1R1* expression does not correlate with markers of systemic inflammation, which may suggest an enhanced activation of local *IL-1β* signaling, independent of systemic inflammation levels in MCS patients.

Further, the expression patterns of *IL-1R1*, *IL-1RaP* and *IL-33* show a strong correlation, in both ventricles as well as in both sub-groups, which indicates a direct or indirect association of *IL-1* and *IL-33* signaling. This hypothesis is supported by a significant correlation of *IL-R1* and *IL-33* receptor, *IL-1RL1*, in all conditions. *IL-1β* upregulates *IL-33* in vitro in cardiac myocytes, cardiac fibroblasts and vascular smooth muscle cells [24], while *IL-1β* stimulation of endothelial cells leads to downregulation of *IL-33* expression [31]. In local immune response, *IL-1β* acts as an upstream inducer of *IL-33* and IL-1RL1 [32]. Since both *IL-33* and *IL-1* signaling are promising targets for drug therapy affecting inflammation-driven fibrotic remodeling of myocardial tissue, a putative link between *IL-33* and *IL-1* signaling requires further exploration.

Comparison of MCS patients with HF patients in this study showed that these two patient groups in part required modification in pharmacologic therapy due to LVAD implantation, e.g., more antiplatelet drugs, PDE5i and calcium antagonists but less antiarrhythmic therapy. Here, medication may additionally affect local signaling, since several studies report the influence of these agents on *IL-1* signaling [33,34]) or *IL-33* signaling [35,36]. Our study provides insight into the topographic differences between the LV and RV myocardia. Expression levels of *IL-1RL1* and of *IL-33* (for MCS) are significantly higher in the RV, which may suggest an increased activation of *IL-33* signaling. However, MCS does not lead to significantly altered *IL-33* expression levels, which is consistent with previous findings [30]. Likewise, in our study, *IL-1RL1* levels do not significantly change due to MCS, in contrast to a report by Caselli et al. [37]. This could be due to the different mean duration of LVAD support or due to the relatively small group size (Caselli et al. studied 7 HF patients versus 6 patients after LVAD support). Therefore, elevated expression within the RV is likely due to topographic differences between ventricles. Various in vitro studies show that *IL-33* and IL-1RL1 expression in the heart impact cardiac remodeling, with improved cardiac function [14,16,38], and *IL-33* / IL-1RL1 signaling may be enhanced by medical treatment [36,39]. Therefore, targeting *IL-33* / IL-1RL1 signaling with pharmaceutical therapies may be beneficial to improve RV function. Clinical data [40,41,42] and experimental models of chronic RV pressure overload [43,44] show an association of RV failure with increased pro-inflammatory mediators and infiltrating immune cells in RV tissue. In contrast, *IL-33* RV expression remains unchanged or decreases during RV failure [43,44]. Further, concentrations of *IL-33* are lower in HF patients than in healthy controls, indicating a role in HF progression [45]. Hence, further investigation of topographic features of the RV myocardium may lead to new therapeutic approaches.

Comparing patients with ICM and DCM, our data show significantly higher LV expression levels of *IL-1R1* and *IL-1RaP* in ICM patients, while *IL-1B* expression is comparable in both patient cohorts. Since analyzed myocardial samples are not explicitly picked from the infarct region and patients with acute or recent myocardial infarction (MI) are excluded, these results indicate that the regulation of *IL-1* signaling in ICM may not be limited to the early phase of remodeling after MI. Since *IL-1* signaling plays an important role in MI and the development of ischemic injury [4,46], inhibition of *IL-1* signaling may be a promising strategy not only after MI [47,48,49,50] but also MI-related HF [51]. However, there is no large-scale post-ischemic anti-inflammatory therapeutic strategy successfully translated into clinical practice yet. Our data support the hypothesis that the sensitivity of the LV to *IL-1* may have an impact on the progression of ischemia-derived heart failure, since *IL-1* receptors show enhanced expression in ICM. This theory is supported by a recent study showing that inhibition of *IL-1β* starting at an extended time-point after reperfusion results in improved systolic function in an ischemia-reperfusion rat model, with already established cardiac dilation and dysfunction [52]. Therefore, inhibition of *IL-1* signaling may be a promising therapeutic strategy for heart failure patients, even with a considerable delay since the index MI event.

Taken together, our results show that MCS is associated with specific changes in *IL-1* and *IL-33* receptor expression and the correlation of receptor expression patterns, indicating an impact of MCS on local signaling in ventricular tissue. The majority of studies focuses on plasma levels of cytokines or soluble receptors, not taking into account local expression changes on the myocardial tissue level [3,19,25]. On the other hand, a number of in vitro studies have analyzed *IL-1* and *IL-33* signaling in the context of HF, hinting at promising new therapeutic approaches aiming at enhancing *IL-33* signaling [36,53,54] or inhibiting *IL-1* signaling, e.g., with neutralizing antibodies. Therefore, analysis of local cytokine receptor expression levels serves as a link between observation in patients and in vitro studies. Further investigations should aim at cell-type-specific analysis of receptor expression to provide a deeper insight into the regulation of *IL-1* and *IL-33* signaling within patients.

## 5. Strengths and Limitations of the Study

The comparatively large patient cohort is one of the strengths of the present study. Moreover, the standardized sample recovery by a trained team, in combination with a single-center approach, provides data from a coherent tissue collection. However, some limitations warrant further consideration when evaluating the results of this study. First, the presented data mirror a single time-point picture of gene expression levels, at the time of transplantation. A longitudinal analysis, possibly comprising myocardial specimens derived from endomyocardial biopsies or LV apex tissue obtained during LVAD implantation, may provide further insight into the dynamics of myocardial expression. Further mRNA expression levels are obtained from whole myocardial tissue samples, therefore not allowing for a detailed specification of cell-type-specific expression. Moreover, it should be noted that causality cannot be necessarily concluded from the significant correlations observed. Moreover, analysis of blood samples at transplantation was not possible in this study because of the lack of respective samples in our biobank. Since device therapy required modification in the pharmacologic therapy of LVAD patients, this in turn may affect the expression of the herein analyzed genes.

## 6. Conclusions

HF and MCS are accompanied by elevated systemic inflammation, however regulation of cytokine receptors within the heart remains elusive. Our results indicate an enhanced local *IL-1β* signaling in patients after long term MCS. The correlating expression of the *IL-1* receptor and *IL-33* may hint at a crosslink between *IL-33* and *IL-1β* signaling. Furthermore, some receptors show significantly higher expression in the RV, indicating topographic differences in *IL-1β* and *IL-33* signaling. The higher expression of *IL-1β* receptors, particularly in ICM, leads to the hypothesis that enhanced *IL-1β* signaling may play a role in remodeling even over an extended period after MI. Both *IL-33* and *IL-1* signaling pathways are promising drug therapy targets for inflammation-driven fibrotic remodeling, therefore further exploration of local *IL-33* and *IL-1* signaling is required.

## Figures and Tables

**Table 1:** Baseline characteristics.

	HF (*n* = 24)	MCS (*n* = 39)	*p*-Value
Gender (male), *n* (%)	17 (70.8%)	33 (84.6%)	n.s.
Age (years)	53 ± 2.2	55 ± 1.8	n.s.
BMI	24.58 ± 1.36	27.90 ± 0.92	n.s.
NYHA Class IV	0 (0%)	4 (10.3%)	0.001
III	2 (8.3%)	18 (46.2%)	
II	18 (75.0%)	16 (41.0%)	
I	4 (16.7%)	1 (2.6%)	
LVEF %	26 ± 2.1	22 ± 4.5	n.s.
DCM, *n* (%)	18 (75%)	20 (51.3%)	n.s.
ICM, *n* (%)	6 (25%)	19 (48.7%)	n.s.
Comorbidities			
Diabetes, *n* (%)	8 (33.3%)	13 (33.3%)	n.s.
Hypertension, *n* (%)	9 (37.5%)	21 (53.8%)	n.s.
Dyslipidemia, *n* (%)	8 (33.3%)	8 (20.5%)	n.s.
Kidney disease, *n* (%)	14 (58.3%)	22 (56.4%)	n.s.
History of smoking, *n* (%)	2 (8.3%)	3 (7.7%)	n.s.
Pre OP laboratory values			
NT-proBNP (pg/mL)	6674 ± 1580	2304 ± 883	0.002
Bilirubin (mg/dL)	1.2 ± 0.26	0.9 ± 0.12	n.s.
Creatinine (mg/dL)	1.7 ± 0.43	1.2 ± 0.08	n.s.
Leucocytes (1000/µL)	9.4 ± 0.84	8.2 ± 0.47	n.s.
CRP (mg/dL)	3.2 ± 1.6	2.1 ± 0.43	n.s.
**Treatments**			
ACE-I and/or ARB, *n* (%)	17 (70.8%)	18 (46.2%)	n.s.
Beta blockers, *n* (%)	16 (66.7%)	34 (87.2%)	n.s.
Statins, *n* (%)	10 (41.6%)	20 (51.3%)	n.s.
Antiplatelet agents, *n* (%)	9 (37.5%)	32 (82.1%)	0.001
Inotropic support, *n* (%)	2 (8.3%)	0 (0%)	n.s.
MR / Aldosterone antagonists, *n* (%)	13 (54.2%)	28 (71.8%)	n.s.
Other diuretics, *n* (%)	19 (79.2%)	27 (69.2%)	n.s.
Antiarrhythmic therapy, *n* (%)	12 (50%)	8 (20.5%)	0.025
Antidiabetic agents, *n* (%)	2 (8.3%)	7 (17.9%)	n.s.
Calcium antagonists, *n* (%)	1 (4.2%)	10 (25.6%)	0.040
PDE5i, *n* (%)	4 (10.3%)	22 (56.4%)	0.003
Allopurinol, *n* (%)	6 (26.7%)	7 (25%)	n.s.

Values are presented as mean ± standard error of mean or as n (percentage). Abbreviations: BMI—body mass index; NYHA class—New York Heart Association class; LVEF—left ventricular ejection fraction; DCM—dilated cardiomyopathy; ICM—ischemic cardiomyopathy; NT-proBNP—N-terminal pro brain natriuretic peptide; CRP—C-reactive protein, ACE-I—angiotensin-converting-enzyme inhibitor; MCR—mineralocorticoid receptor, PDE5i—phosphodiesterase-5 inhibitor.

**Table 2:** Strong correlation between *IL-1R1*, *IL-1RaP* and *IL-33*.

**r-Value**						
**HF**		**LV**			**RV**	
	*IL1RaP*	*IL1RL1*	*IL-33*	*IL1RaP*	*IL1RL1*	*IL-33*
*IL1R1*	**0.73**	**0.48**	**0.93**	**0.79**	**0.75**	**0.78**
*IL1RaP*		**0.46**	**0.75**		**0.68**	**0.77**
*IL1RL1*			0.32			**0.58**
**MCS**						
*IL1R1*	**0.83**	**0.46**	**0.78**	**0.75**	**0.36**	**0.65**
*IL1RaP*		0.29	**0.74**		0.29	**0.57**
*IL1RL1*			0.22			0.02
***p*-Value**						
**HF**		**LV**			**RV**	
	*IL1RaP*	*IL1RL1*	*IL-33*	*IL1RaP*	*IL1RL1*	*IL-33*
*IL1R1*	<0.0001	0.0163	<0.0001	<0.0001	<0.0001	<0.0001
*IL1RaP*		0.0248	<0.0001		0.0003	<0.0001
*IL1RL1*			n.s.			0.0031
**MCS**						
*IL1R1*	<0.0001	0.0028	<0.0001	<0.0001	0.0224	<0.0001
*IL1RaP*		n.s. (0.073)	<0.0001		n.s. (0.078)	0.0001
*IL1RL1*			n.s.			n.s.

Spearman correlations are calculated analyzing mRNA expression levels of *IL-1R1*, *IL-1RaP*, *IL-1RL1* and *IL-33*. Correlation coefficient r is displayed in the upper part of the table, and significant correlation is indicated with bold formatting, *p*-values are listed in the lower part.

**Table 3:** Local expression levels correlate with CRP and leucocyte serum levels.

**r**	**HF**		**MCS**		***p***	**HF**		**MCS**	
	**LV**	**RV**	**LV**	**RV**		**LV**	**RV**	**LV**	**RV**
IL-1B	−0.15	0.05	0.00	−0.06		n.s.	n.s.	n.s.	n.s.
IL-1R1	**0.56**	0.41	0.32	−0.06		0.0067	n.s. (0.059)	n.s. (0.053)	n.s.
IL-1R2	0.10	0.11	0.32	0.08		n.s.	n.s.	n.s. (0.061)	n.s.
IL-1RaP	0.27	0.18	0.15	−0.18		n.s.	n.s.	n.s.	n.s.
IL-1RL1	0.23	**0.44**	**0.34**	0.17		n.s.	0.0421	0.0402	n.s.
*IL-33*	**0.58**	**0.48**	0.19	−0.06		0.0041	0.0239	n.s.	n.s.
**Serum leucocyte number**									
**r**	**HF**		**MCS**		***p***	**HF**		**MCS**	
	**LV**	**RV**	**LV**	**RV**		**LV**	**RV**	**LV**	**RV**
IL-1B	0.01	0.15	0.19	0.05		n.s.	n.s.	n.s.	n.s.
IL-1R1	**0.52**	0.05	0.18	0.02		0.0097	n.s.	n.s.	n.s.
IL-1R2	0.11	−0.20	0.17	0.10		n.s.	n.s.	n.s.	n.s.
IL-1RaP	0.12	−0.12	−0.03	0.04		n.s.	n.s.	n.s.	n.s.
IL-1RL1	0.35	0.06	0.16	0.09		n.s. (0.09)	n.s.	n.s.	n.s.
*IL-33*	**0.46**	0.07	−0.15	0.05		0.0242	n.s.	n.s.	n.s.

*IL-1B*, *IL-1R1*, *IL-1R2*, *IL-1RaP*, *IL-1RL1* and *IL-33* expression levels are correlated with CRP levels and leucocyte count. Spearman correlation coefficient r (right) is indicated in bold formatting for significant correlations and *p*-values are listed (left).

## Data Availability

The data that support the findings of this study are available from the corresponding author upon reasonable request.

## Supplementary Materials

The following are available online at https://www.mdpi.com/article/10.3390/jcm10214856/s1, Figure S1: selection criteria of myocardial tissue samples, Table S1: baseline characteristics of patients with DCM versus ICM; Figure S2: validation results of real time PCR.

## References

[1] McDonagh T.A., Metra M., Adamo M., Gardner R.S., Baumbach A., Böhm M., Burri H., Butler J., Čelutkienė J., Chioncel O. (2021). 2021 ESC Guidelines for the diagnosis and treatment of acute and chronic heart failure. Eur. Heart J..

[2] Frigerio M. (2021). Left Ventricular Assist Device: Indication, Timing, and Management. Heart Fail. Clin..

[3] Grosman-Rimon L., Billia F., Fuks A., Jacobs I., McDonald M.A., Cherney D.Z., Rao V., Billia F. (2016). New therapy, new challenges: The effects of long-term continuous flow left ventricular assist device on inflammation. Int. J. Cardiol..

[4] Kawaguchi M., Takahashi M., Hata T., Kashima Y., Usui F., Morimoto H., Izawa A., Takahashi Y., Masumoto J., Koyama J. (2011). Inflammasome Activation of Cardiac Fibroblasts Is Essential for Myocardial Ischemia/Reperfusion Injury. Circulation.

[5] Bujak M., Dobaczewski M., Chatila K., Mendoza L.H., Li N., Reddy A., Frangogiannis N. (2008). Interleukin-1 Receptor Type I Signaling Critically Regulates Infarct Healing and Cardiac Remodeling. Am. J. Pathol..

[6] Eriksson U., Kurrer M.O., Sonderegger I., Iezzi G., Tafuri A., Hunziker L., Suzuki S., Bachmaier K., Bingisser R., Penninger J. (2003). Activation of Dendritic Cells through the Interleukin 1 Receptor 1 Is Critical for the Induction of Autoimmune Myocarditis. J. Exp. Med..

[7] Francis S., Holden H., Holt C.M., Duff G.W. (1998). Interleukin-1 in Myocardium and Coronary Arteries of Patients with Dilated Cardiomyopathy. J. Mol. Cell. Cardiol..

[8] Humeres C., Frangogiannis N.G. (2019). Fibroblasts in the Infarcted, Remodeling, and Failing Heart. JACC Basic Transl. Sci..

[9] Saxena A., Chen W., Su Y., Rai V., Uche O.U., Li N., Frangogiannis N. (2013). *IL-1* Induces Proinflammatory Leukocyte Infiltration and Regulates Fibroblast Phenotype in the Infarcted Myocardium. J. Immunol..

[10] Segiet O.A. (2019). The role of interleukins in heart failure with reduced ejection fraction. Anatol. J. Cardiol..

[11] Barton P.J., Birks E.J., Felkin L., Cullen E.M., Koban M.U., Yacoub M.H. (2003). Increased expression of extracellular matrix regulators TIMP1 and MMP1 in deteriorating heart failure. J. Heart Lung Transplant..

[12] Bedi M.S., Alvarez R.J., Kubota T., Sheppard R., Kormos R.L., Siegenthaler M.P., Feldman A.M., McTiernan C.F., McNamara D.M. (2008). Myocardial Fas and Cytokine Expression in End-Stage Heart Failure: Impact of LVAD Support. Clin. Transl. Sci..

[13] Garlanda C., Dinarello C.A., Mantovani A. (2013). The Interleukin-1 Family: Back to the Future. Immunity.

[14] Veeraveedu P.T., Sanada S., Okuda K., Fu H.Y., Matsuzaki T., Araki R., Yamato M., Yasuda K., Sakata Y., Yoshimoto T. (2017). Ablation of *IL-33* gene exacerbate myocardial remodeling in mice with heart failure induced by mechanical stress. Biochem. Pharmacol..

[15] Sanada S., Hakuno D., Higgins L.J., Schreiter E., McKenzie A.N., Lee R.T. (2007). *IL-33* and ST2 comprise a critical biomechanically induced and cardioprotective signaling system. J. Clin. Investig..

[16] Abston E.D., Barin J.G., Cihakova D., Bucek A., Coronado M.J., Brandt J.E., Bedja D., Kim J.B., Georgakopoulos D., Gabrielson K.L. (2012). *IL-33* Independently Induces Eosinophilic Pericarditis and Cardiac Dilation. Circ. Heart Fail..

[17] Chen W.-Y., Hong J., Gannon J., Kakkar R., Lee R.T. (2015). Myocardial pressure overload induces systemic inflammation through endothelial cell IL-33. Proc. Natl. Acad. Sci. USA.

[18] Schmitz J., Owyang A., Oldham E., Song Y., Murphy E., McClanahan T.K., Zurawski G., Moshrefi M., Qin J., Li X. (2005). IL-33, an Interleukin-1-like Cytokine that Signals via the *IL-1* Receptor-Related Protein ST2 and Induces T Helper Type 2-Associated Cytokines. Immunity.

[19] Díez J., Bayes-Genis A. (2017). Compelling Benefit of Soluble Suppression of Tumorigenicity-2 in Post–Myocardial Infarction Estimation of Risk: The Time Is Right for Its Routine Use in the Clinic. J. Am. Heart Assoc..

[20] Zhang T., Xu C., Zhao R., Cao Z. (2021). Diagnostic Value of sST2 in Cardiovascular Diseases: A Systematic Review and Meta-Analysis. Front. Cardiovasc. Med..

[21] Bi J., Garg V., Yates A. (2021). Galectin-3 and sST2 as Prognosticators for Heart Failure Requiring Extracorporeal Life Support: Jack n’ Jill. Biomolecules.

[22] Tseng C.C.S., Huibers M.M.H., Van Kuik J., De Weger R.A., Vink A., De Jonge N. (2017). The Interleukin-33/ST2 Pathway Is Expressed in the Failing Human Heart and Associated with Pro-fibrotic Remodeling of the Myocardium. J. Cardiovasc. Transl. Res..

[23] Bartunek J., Delrue L., Van Durme F., Muller O., Casselman F., De Wiest B., Croes R., Verstreken S., Goethals M., de Raedt H. (2008). Nonmyocardial Production of ST2 Protein in Human Hypertrophy and Failure Is Related to Diastolic Load. J. Am. Coll. Cardiol..

[24] Demyanets S., Kaun C., Pentz R., Krychtiuk K., Rauscher S., Pfaffenberger S., Zuckermann A., Aliabadi A., Gröger M., Maurer G. (2013). Components of the interleukin-33/ST2 system are differentially expressed and regulated in human cardiac cells and in cells of the cardiac vasculature. J. Mol. Cell. Cardiol..

[25] Tseng C.C.S., Huibers M.M.H., Gaykema L.H., Koning E.S.-D., Ramjankhan F.Z., Maisel A.S., de Jonge N. (2018). Soluble ST2 in end-stage heart failure, before and after support with a left ventricular assist device. Eur. J. Clin. Investig..

[26] Aleksova A., Beltrami A.P., Carriere C., Barbati G., Lesizza P., Perrieri-Montanino M., Isola M., Gentile P., Salvioni E., Not T. (2017). Interleukin-1β levels predict long-term mortality and need for heart transplantation in ambulatory patients affected by idiopathic dilated cardiomyopathy. Oncotarget.

[27] Szekely Y., Arbel Y. (2018). A Review of Interleukin-1 in Heart Disease: Where Do We Stand Today?. Cardiol. Ther..

[28] Vieira J.L., Ventura H.O., Mehra M.R. (2020). Mechanical circulatory support devices in advanced heart failure: 2020 and beyond. Prog. Cardiovasc. Dis..

[29] Vanderheyden M., Paulus W., Voss M., Knuefermann P., Sivasubramanian N., Mann D., Baumgarten G. (2005). Myocardial cytokine gene expression is higher in aortic stenosis than in idiopathic dilated cardiomyopathy. Heart.

[30] Birks E.J., Latif N., Owen V., Bowles C., Felkin L.E., Mullen A.J., Khaghani A., Barton P.J., Polak J.M., Pepper J.R. (2001). Quantitative Myocardial Cytokine Expression and Activation of the Apoptotic Pathway in Patients Who Require Left Ventricular Assist Devices. Circulation.

[31] Küchler A.M., Pollheimer J., Balogh J., Sponheim J., Manley L., Sorensen D.R., De Angelis P.M., Scott H., Haraldsen G. (2008). Nuclear Interleukin-33 Is Generally Expressed in Resting Endothelium but Rapidly Lost upon Angiogenic or Proinflammatory Activation. Am. J. Pathol..

[32] Ho J.E., Chen W.-Y., Chen M.-H., Larson M.G., McCabe E.L., Cheng S., Ghorbani A., Coglianese E., Emilsson V., Johnson A.D. (2013). Common genetic variation at the IL1RL1 locus regulates IL-33/ST2 signaling. J. Clin. Investig..

[33] Lu J., Liu F., Chen F., Jin Y., Chen H., Liu D., Cui W. (2016). Amlodipine and atorvastatin improve ventricular hypertrophy and diastolic function via inhibiting TNF-α, *IL-1β* and NF-κB inflammatory cytokine networks in elderly spontaneously hypertensive rats. Biomed. Pharmacother..

[34] Zahran M.H., Hussein A.M., Barakat N., Awadalla A., Khater S., Harraz A., Shokeir A.A. (2015). Sildenafil activates antioxidant and antiapoptotic genes and inhibits proinflammatory cytokine genes in a rat model of renal ischemia/reperfusion injury. Int. Urol. Nephrol..

[35] Zhang L., Hu M., Chen Y., Wang Y. (2020). Effects of atorvastatin and ticagrelor combination therapy on renal function and the levels of suppression of tumorigenicity 2 and interleukin-33 in patients with ST-segment elevation myocardial infarction. J. Int. Med Res..

[36] Xia J., Qu Y., Yin C., Xu D. (2017). Preliminary study of beta-blocker therapy on modulation of interleukin-33/ST2 signaling during ventricular remodeling after acute myocardial infarction. Cardiol. J..

[37] Caselli C., D’Amico A., Ragusa R., Caruso R., Prescimone T., Cabiati M., Nonini S., Marraccini P., Del Ry S., Trivella M.G. (2013). IL-33/ST2 Pathway and Classical Cytokines in End-Stage Heart Failure Patients Submitted to Left Ventricular Assist Device Support: A Paradoxic Role for Inflammatory Mediators?. Mediat. Inflamm..

[38] Sánchez-Más J., Lax A., Asensio-López M.D.C., Palacio M.J.F.-D., Caballero L., Santarelli G., Januzzi J.L., Figal D.A.P. (2014). Modulation of IL-33/ST2 system in postinfarction heart failure: Correlation with cardiac remodelling markers. Eur. J. Clin. Investig..

[39] Lax A.M., Sanchez-Mas J., Asensio-Lopez M.C., Palacio M.J.F.-D., Caballero L., Garrido I.P., Pastor-Perez F.J., Januzzi J.L., Pascual-Figal D.A. (2015). Mineralocorticoid Receptor Antagonists Modulate Galectin-3 and Interleukin-33/ST2 Signaling in Left Ventricular Systolic Dysfunction After Acute Myocardial Infarction. JACC Heart Fail..

[40] Dewachter L., Dewachter C. (2018). Inflammation in Right Ventricular Failure: Does It Matter?. Front. Physiol..

[41] Begieneman M.P.V., van de Goot F.R.W., Bilt I.A.C.V.D., Noordegraaf A.V., Spreeuwenberg M.D., Paulus W.J., van Hinsbergh V.W.M., Visser F.C., Niessen H.W.M. (2007). Pulmonary embolism causes endomyocarditis in the human heart. Heart.

[42] Frustaci A., Petrosillo N., Vizza D., Francone M., Badagliacca R., Verardo R., Fedele F., Ippolito G., Chimenti C. (2014). Myocardial and microvascular inflammation/infection in patients with HIV-associated pulmonary artery hypertension. AIDS.

[43] Belhaj A., Dewachter L., Kerbaul F., Brimioulle S., Dewachter C., Naeije R., Rondelet B. (2013). Heme Oxygenase-1 and Inflammation in Experimental Right Ventricular Failure on Prolonged Overcirculation-Induced Pulmonary Hypertension. PLoS ONE.

[44] Dewachter C., Belhaj A., Rondelet B., Vercruyssen M., Schraufnagel D.P., Remmelink M., Brimioulle S., Kerbaul F., Naeije R., Dewachter L. (2015). Myocardial inflammation in experimental acute right ventricular failure: Effects of prostacyclin therapy. J. Hear. Lung Transplant..

[45] Segiet O.A. (2019). The concentration of interleukin-33 in heart failure with reduced ejection fraction. Anatol. J. Cardiol..

[46] Pomerantz B.J., Reznikov L.L., Harken A.H., Dinarello C.A. (2001). Inhibition of caspase 1 reduces human myocardial ischemic dysfunction via inhibition of IL-18 and IL-1. Proc. Natl. Acad. Sci. USA.

[47] Abbate A., Salloum F., Van Tassell B.W., Vecile E., Toldo S., Seropian I., Mezzaroma E., Dobrina A. (2011). Alterations in the Interleukin-1/Interleukin-1 Receptor Antagonist Balance Modulate Cardiac Remodeling following Myocardial Infarction in the Mouse. PLoS ONE.

[48] Sager H.B., Heidt T., Hulsmans M., Dutta P., Courties G., Sebas M., Wojtkiewicz G., Tricot B., Iwamoto Y., Sun Y. (2015). Targeting Interleukin-1β Reduces Leukocyte Production After Acute Myocardial Infarction. Circulation.

[49] Panahi M., Papanikolaou A., Torabi A., Zhang J.-G., Khan H., Vazir A., Hasham M., Cleland J.G.F., Rosenthal N., Harding S. (2018). Immunomodulatory interventions in myocardial infarction and heart failure: A systematic review of clinical trials and meta-analysis of *IL-1* inhibition. Cardiovasc. Res..

[50] Abbate A., Trankle C.R., Buckley L.F., Lipinski M.J., Appleton D., Kadariya D., Canada J.M., Carbone S., Roberts C.S., Abouzaki N. (2020). Interleukin-1 Blockade Inhibits the Acute Inflammatory Response in Patients With ST-Segment–Elevation Myocardial Infarction. J. Am. Heart Assoc..

[51] Everett B.M., Cornel J., Lainscak M., Anker S.D., Abbate A., Thuren T., Libby P., Glynn R.J., Ridker P.M. (2019). Anti-Inflammatory Therapy with Canakinumab for the Prevention of Hospitalization for Heart Failure. Circulation.

[52] Harouki N., Nicol L., Remy-Jouet I., Henry J.-P., Dumesnil A., Lejeune A., Renet S., Golding F., Djerada Z., Wecker D. (2017). The *IL-1β* Antibody Gevokizumab Limits Cardiac Remodeling and Coronary Dysfunction in Rats with Heart Failure. JACC Basic Transl. Sci..

[53] Van Tassell B.W., Abouzaki N.A., Erdle C.O., Carbone S., Trankle C.R., Melchior R.D., Turlington J.S., Thurber C.J., Christopher S., Dixon D.L. (2016). Interleukin-1 Blockade in Acute Decompensated Heart Failure. J. Cardiovasc. Pharmacol..

[54] Ridker P.M., Everett B.M., Thuren T., MacFadyen J.G., Chang W.H., Ballantyne C., Fonseca F., Nicolau J., Koenig W., Anker S.D. (2017). Antiinflammatory Therapy with Canakinumab for Atherosclerotic Disease. N. Engl. J. Med..

