# Supplementary material for: Altered mRNA Expression of Interleukin-1 Receptors in Myocardial Tissue of Patients with Left Ventricular Assist Device Support
Source: J Clin Med. 2021 Oct 22;10(21):4856. doi: 10.3390/jcm10214856 (PMC8584390; doi:10.3390/jcm10214856)
Supplement: Supplementary file 1 [file jcm-10-04856-s001.zip › Legends S1.pdf]

Journal of
Clinical Medicine

MDPI

Supplementary Legends

Figure S1: Selection criteria of myocardial tissue samples. From 101 patients undergoing HTx between August 2011 and December 2018, 38 patients were excluded due to very short LVAD support (n=11), heart failure causes other than ischemic (ICM) and non-ischemic dilated cardiomyopathy (DCM) or mixed genesis (n=13), previously otherwise analyzed myocardial tissue samples(n=13) and diagnosed infectious disease presenting a hazard to the laboratory team (n=1).

Table S1: Baseline characteristics of patients with DCM versus ICM Values are presented as mean ± standard error of means or as n (percentage). Abbreviations: DCM - in dilated cardiomyopathy; ICM - ischemic cardiomyopathy; BMI - body mass index; NYHA Class - New York Heart Association Class; LVEF - left ventricular ejection fraction; NT-proBNP - N-terminal pro brain natriuretic peptide; CRP - C-reactive protein, ACE-I - angiotensin-converting-enzyme inhibitor; MCR - mineralocorticoid receptor, PDE5i - phosphodiesterase-5 inhibitor.
