# Supplementary material for: Altered mRNA Expression of Interleukin-1 Receptors in Myocardial Tissue of Patients with Left Ventricular Assist Device Support
Source: J Clin Med. 2021 Oct 22;10(21):4856. doi: 10.3390/jcm10214856 (PMC8584390; doi:10.3390/jcm10214856)
Supplement: Supplementary file 1 [file jcm-10-04856-s001.zip › Table S1.pdf]

Journal of
Clinical Medicine

	DCM (n=38)	ICM (n=25)	p-value
Gender (male), n (%)	30 (78.9%)	20 (80.0%)	n.s.
Age (years)	50 ± 1.8	60 ± 1.4	0.02
BMI	27.13 ± 0.95	26 ± 1.4	n.s.
NYHA Class IV	1 (2.6%)	3 (8.0%)	0.014
III	9 (23.7%)	11 (44.0%)	
II	24 (63.2%)	10 (40.0%)	
I	4 (10.5%)	1 (4.0%)	
LVEF %	22 ± 1.1	25 ± 2.0	n.s.
LVAD, n (%)	20 (52.6%)	19 (76.0%)	n.s.
**Comorbidities**			
Diabetes, n (%)	6 (15.8%)	15 (60.0%)	0.000
Hypertension, n (%)	14 (36.8%)	16 (64.0%)	0.044
Dyslipidemia, n (%)	9 (23.7%)	7 (28.0%)	n.s.
Kidney Disease, n (%)	21 (55.3%)	15 (60.0%)	n.s.
History of smoking, n (%)	3 (7.9%)	5 (20.0%)	n.s.
**Pre OP laboratory values**			
NT-proBNP (pg/ml)	4662 ± 1245	3525 ± 1325	n.s.
Bilirubin (mg/dL)	1.1 ± 0.14	1.0 ± 0.25	n.s.
Creatinine (mg/dL)	1.6 ± 0.29	1.3 ± 0.10	n.s.
Leucocytes (1000/µl)	8.6 ± 0.6	8.9 ± 0.6	n.s.
CRP (mg/dL)	1.4 ± 0.3	4.1 ± 1.5	n.s.
**Treatments**			
ACE-I and/or ARB, n (%)	25 (65.7%)	10 (40.0%)	n.s.
Beta blockers, n (%)	29 (76.3%)	21 (84.0%)	n.s.
Statins, n (%)	13 (34.2%)	17 (68.0%)	0.011
Antiplatelet agents, n (%)	20 (52.6%)	21 (84.0%)	n.s.
Inotropic support, n (%)	2 (5.3%)	0 (0%)	n.s.
MR / Aldosterone antagonists, n (%)	24 (63.2%)	17 (68.0%)	n.s.
Other diuretics, n (%)	28 (73.7%)	18 (72.0%)	n.s.
Antiarrhythmic therapy, n (%)	16 (42.1%)	4 (16.0%)	n.s.
antidiabetic agents, n (%)	2 (5.3%)	7 (28.0%)	0.023
Calcium antagonists, n (%)	4 (10.5%)	7 (28.0%)	n.s.
PDE5i, n (%)	12 (31.5%)	14 (56.0%)	n.s.
Allopurinol, n (%)	8 (21.1%)	5 (20.0%)	n.s.
