# Supplementary figures and images for: Altered mRNA Expression of Interleukin-1 Receptors in Myocardial Tissue of Patients with Left Ventricular Assist Device Support
Source: J Clin Med. 2021 Oct 22;10(21):4856. doi: 10.3390/jcm10214856 (PMC8584390; doi:10.3390/jcm10214856)

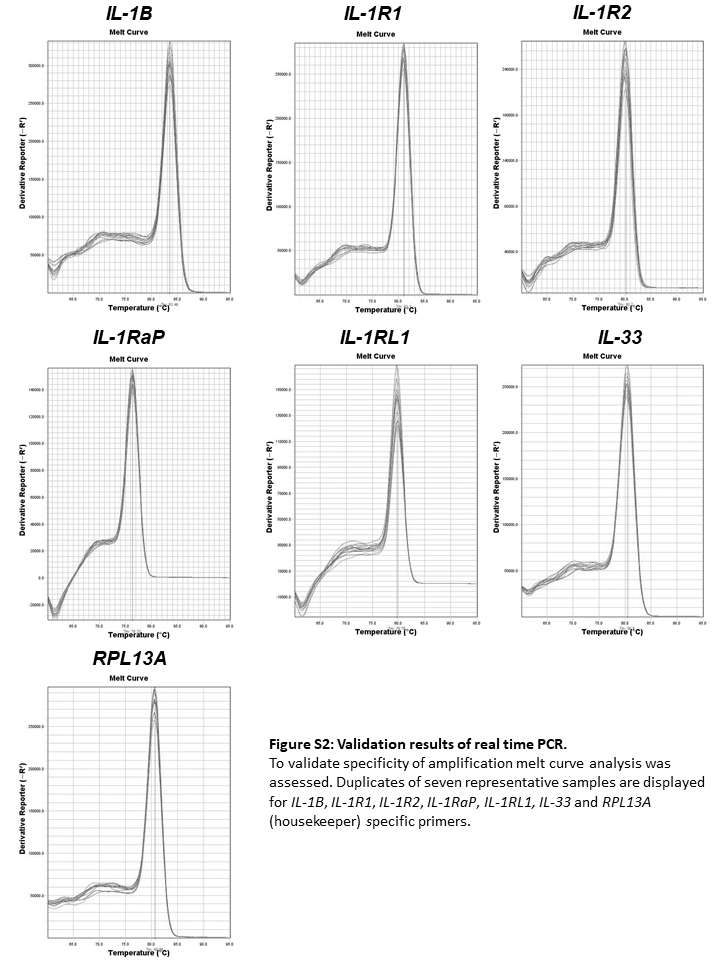

Supplement: Supplementary file 1 [file jcm-10-04856-s001.zip › Figure S2_Revision.tif]

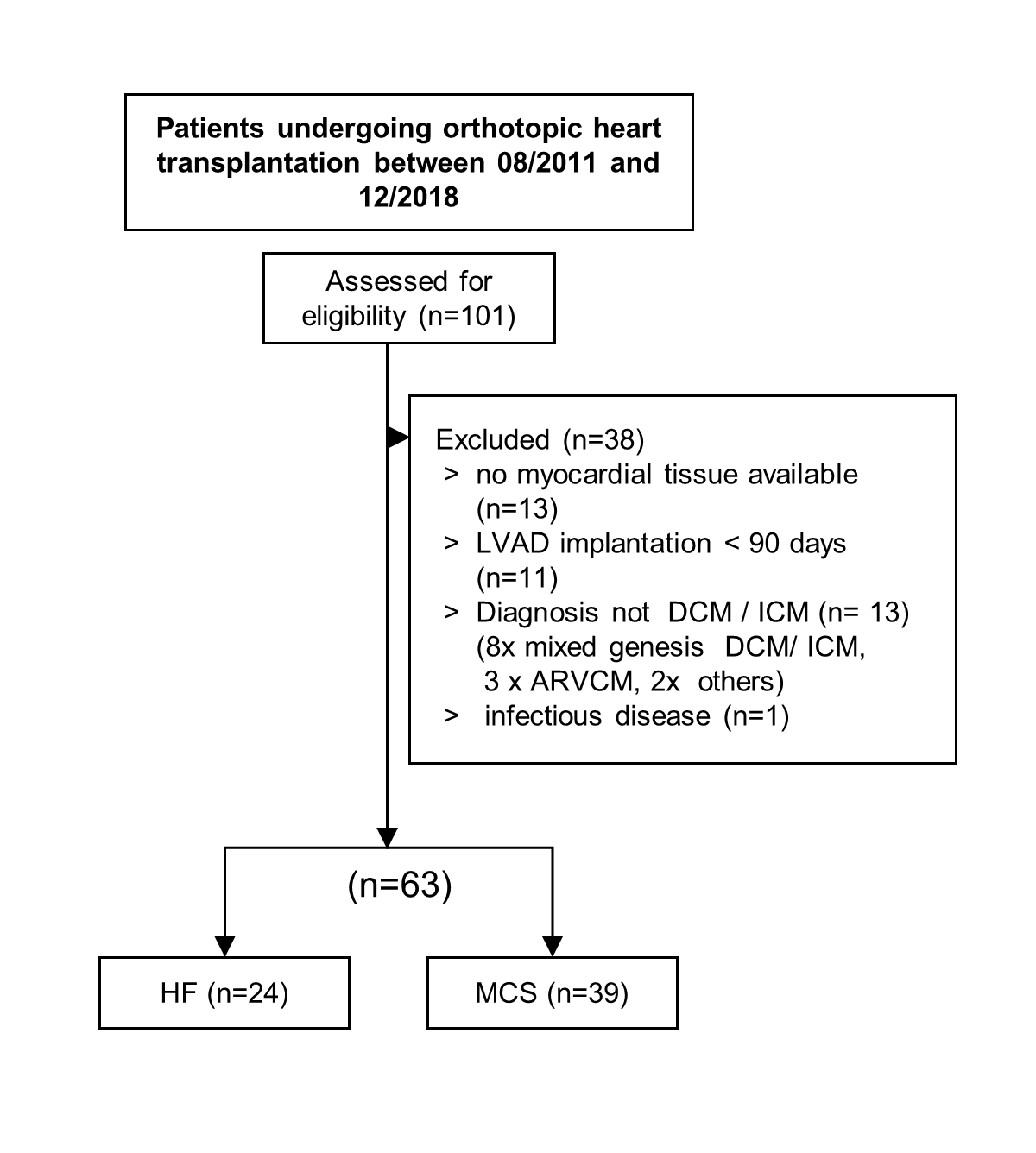

Supplement: Supplementary file 1 [file jcm-10-04856-s001.zip › FigureS1.tif]
